# Mesenchymal Stem Cells: New Players in Retinopathy Therapy

**DOI:** 10.3389/fendo.2014.00059

**Published:** 2014-04-24

**Authors:** Gangaraju Rajashekhar

**Affiliations:** ^1^Indiana Center for Vascular Biology and Medicine, Indiana University School of Medicine, Indianapolis, IN, USA; ^2^Eugene and Marilyn Glick Eye Institute, Indiana University School of Medicine, Indianapolis, IN, USA; ^3^Vascular and Cardiac Center for Adult Stem Cell Therapy, Indiana University School of Medicine, Indianapolis, IN, USA; ^4^VA Center for Regenerative Medicine, Indiana University School of Medicine, Indianapolis, IN, USA; ^5^Department of Ophthalmology, Indiana University School of Medicine, Indianapolis, IN, USA; ^6^Department of Cellular and Integrative Physiology, Indiana University School of Medicine, Indianapolis, IN, USA

**Keywords:** adult stem cells, pericyte, ASC, EPC, clinical trial, neurodegeneration, ERG

## Abstract

Retinopathies in human and animal models have shown to occur through loss of pericytes resulting in edema formation, excessive immature retinal angiogenesis, and neuronal apoptosis eventually leading to blindness. In recent years, the concept of regenerating terminally differentiated organs with a cell-based therapy has evolved. The cells used in these approaches are diverse and include tissue-specific endogenous stem cells, endothelial progenitor (EPC), embryonic stem cells, induced pluripotent stem cells (iPSC) and mesenchymal stem cells (MSC). Recently, MSC derived from the stromal fraction of adipose tissue have been shown to possess pluripotent differentiation potential *in vitro*. These adipose stromal cells (ASC) have been differentiated in a number of laboratories to osteogenic, myogenic, vascular, and adipocytic cell phenotypes. *In vivo*, ASC have been shown to have functional and phenotypic overlap with pericytes lining microvessels in adipose tissues. Furthermore, these cells either in paracrine mode or physical proximity with endothelial cells, promoted angiogenesis, improved ischemia–reperfusion, protected from myocardial infarction, and were neuroprotective. Owing to the easy isolation procedure and abundant supply, fat-derived ASC are a more preferred source of autologous mesenchymal cells compared to bone marrow MSC. In this review, we present evidence that these readily available ASC from minimally invasive liposuction will facilitate translation of ASC research into patients with retinal diseases in the near future.

## Introduction

Diabetic retinopathy (DR) is the most common vascular complication in patients with long-standing diabetes, and is the leading cause of blindness in working-age adults. The estimated prevalence in USA is 5.4% (~7 million) (1). Future projections suggest that DR will increase as a public health problem, with increase in aged population and increasing prevalence of diabetes over time. In the early stages of DR, one notices clinically significant macular edema (ME). This presumably develops concomitant with pericyte loss, basement membrane thickening, and endothelial dysfunction involving loss of its barrier integrity. Subsequent closure of retinal capillaries results in retinal ischemia and aberrant growth factor secretion, neovascular formation, characteristic of proliferative DR (PDR). Current strategies for the therapeutic management of DR include laser photocoagulation, intravitreal triamcinolone (IVT), and intravitreal injection of VEGF neutralizing agents (e.g., Avastin). Patients undergoing laser treatment continue to remain at risk for new bleeding episode requiring multiple laser treatments. Triamcinolone, which is relatively more successful in suppressing ME, requires intravitreal administration. However, this procedure is fraught not only with side effects but also adverse reactions such as sub-capsular cataract, onset of steroid-induced glaucoma, and potential for endophthalmitis. Use of intravitreal Avastin against ME or PDR has not been successful with the effect lasting for only a short period.

## Role for Pericytes in DR

Diabetic retinopathy develops as sustained metabolic dysregulation and inflicts progressive damage to the retinal microvasculature, increasing vascular permeability, and, in advance stages, leads to the aberrant proliferation of vascular endothelial cells (2). As the global level of diabetes is increasing at a rapid rate, there is necessity to understand the molecular mechanisms responsible for diabetes-induced complications, specifically retinal pathology. One of the well-accepted theories of potential development of DR is due to loss of pericytes (3). Pericytes are specialized perivascular cells, derived from the vascular smooth muscle lineage, that reside in close contact with endothelial cells within a common basement membrane. Although pericytes share many markers with smooth muscle cells (SMC), they can be distinguished from SMC by decreased SMC-alpha-actin and increased platelet-derived growth factor (PDGF)-receptor (PDGFR) expression (3). They are believed to provide a nourishing, anti-inflammatory and anti-angiogenic environment for endothelial cells. The formation of the blood–retinal barrier (BRB) is dependent on the interaction of the vascular endothelial cells with both glial cells and pericytes. Endothelial cells recruit pericytes through expression of PDGF, and pericyte activation, in turn, modulates blood vessel homeostasis and endothelial cell growth. The importance of PDGF signaling has been demonstrated with a loss of function experiments of PDGF signaling, resulting in lower retinal pericytes and subsequent vascular degeneration and increased vascular angiogenesis (4). More recently, a new protein kinase C-δ (PKC-δ)-dependent signaling pathway in pericytes via SHP-1 protein has been shown as a mechanism of pericyte apoptosis by downregulating PDGF signaling (5). Angiopoietin-1 (Ang-1) is one pericyte produced factor believed to be essential for these stabilizing functions (6). Ang-1 has been demonstrated to increase survival, anti-inflammatory properties, and barrier function of endothelial cells and absence of Ang-1 leads to increased expression of Ang-2 (7). Ang-2 is an antagonist of Ang-1, which can interfere with tie-2 receptor activation and signaling, leading to decreased endothelial and pericyte cell survival but also to increased endothelial cell activation (8, 9). Recently, the anti-inflammatory activity of Ang-1 has been explained by decreasing the responsiveness of endothelial cell to TNFα (10). Furthermore, inflammatory cytokines such as TNF has been linked to pericyte apoptosis and inhibition of TNF reduced pericyte ghost formation with decreased acellular capillary formation caused by type 1 and type 2 diabetes (11). Accumulating evidence points to the fact that exposure of retinal pericytes to high glucose results in increased pro-apoptotic Bim expression and oxidative stress leading to reduced migration with a significant impact on their rate of apoptosis (12). These findings implicate pericytes as potential mediators of DR and outline potential strategies for targeted therapies based on their regulation.

## Stem Cells and Occular Angiogenesis

Loss of pericytes and endothelial cells was central to the pathogenesis of DR (2). If cell loss is solely responsible for disease, a definitive treatment would involve cell replacement. Stem cells offer the promise of regenerating tissue in organs such as the eye, brain, and heart, damaged by trauma or disease. Over the past decade, literature has emerged that strongly supports the potential for exploiting stem/progenitor cells to maintain, and support abnormal tissue in several diseases, which perhaps can also be extrapolated to retinal diseases. About four basic populations of cells are known as of today (13) that contain progenitor cells which, under appropriate circumstances, may have therapeutic application in the treatment of retinal disease: (1) retinal stem cells that can give rise to photoreceptors and other retinal neurons; (2) Mueller/glial stem cells that can differentiate into retinal glia and/or neurons; (3) retinal pigment epithelial (RPE) stem cells that can not only serve to replace diseased RPE but perhaps can also be stimulated to differentiate into photoreceptors; and (4) endothelial, myeloid progenitor cells, adult stem cells, induced pluripotent stem cells (iPSC) that can contribute to the retinal vasculature and exert vasculo- and neurotrophic rescue effects (Table 1). Research aimed at re-engineering stem cells to develop into vasculature is of great benefit to the DR patients. One of the early publications from Friedlander’s laboratory reported that Lin^−^ hematopoietic stem cells (HSCs) from bone marrow injected directly into the mouse eye targeted activated astrocytes, and participate in normal developmental angiogenesis in neonatal mice or injury-induced neovascularization in the adult (14, 15). Interestingly, these HSCs, transfected with a plasmid encoding a secreted anti-angiogenic peptide, T2-tryptophanyl-tRNA synthetase, profoundly inhibited retinal angiogenesis suggesting that engineered to produce a secreted peptide that can inhibit further proliferation of new vessels and perhaps to stabilize (mature) vessels, provides the hope that cell-based therapy may be useful in the inhibition of proliferative retinopathies (14). Lineage identification in these HSC suggested that CD44^hi^ cells via a HIF1α-mediated mechanism suggested to play a role in oxygen-induced retinopathy model (OIR) (15). It is noteworthy to mention that in this study, no labeling was present with antibodies against CD31 or NG2 indicating that these cells are unlikely to be differentiating into endothelial cells or pericytes but rather gave neurotrophic support to form neovascularization (13). In contrary, another model of adult neovascularization using organ-specific VEGF expression, Grunewald et al. showed that bone marrow-derived myeloid cells targeted the site of VEGF expression in an SDF-1-mediated mechanism and promoted vessel growth after assuming perivascular localization (16). Along the same lines, another recent study demonstrated that SDF-1 released from platelets mobilized and recruited CD11b-expressing myeloid cells to sites of neovascularization in a model of hind-limb ischemia for new vessel growth (17). Alternate approaches included, insulin-like growth factor binding protein-3 (IGFBP-3) increased differentiation of GFP(+) HSCs into pericytes and astrocytes thereby increasing vascular ensheathment of pericytes and decreasing apoptosis of pericytes and retinal neurons in the OIR model. Among cell-based approaches intended to address DR, in addition to myeloid progenitor cells, intravitreal injection of CD34+ endothelial cells (18) have been very successful to prevent vascular regression and protect neurons in genetic mouse models of retinal degeneration. However, to date, the identity and cell surface markers expressed by these cells remain incompletely defined (19). Diabetes causes metabolic and physiologic abnormalities in the retina, and these changes suggest a role for inflammation, altered cytokine expression (11, 20–28), dysfunction, and even degeneration of some neuronal cells (29, 30). These findings call for potential targeted cell therapies that display anti-inflammatory and anti-apoptotic behavior for subsequent vascular stabilization and neuronal repair early in DR rather than at later stages of vascular angiogenesis. There is also poor understanding of signaling events in these transplanted stem cells. A comprehensive understanding of key cellular and molecular pathways is urgently needed so as to optimize their ability to survive in the pathological *in vivo* diabetic microenvironment. It is imperative that for treatment of diabetic vessel damage, transplanted cells be capable of withstanding the *in vivo* diabetic microenvironment (31).

**Table 1:** **Stem cells that are implicated in the cell-based therapy for retinal diseases**.

Source	Stem/progenitor markers	Reference
Hematopoietic stem cells (HSC)	CD44hi/CD11a+/CD11b+/Ly6G/C+/F4/80+/CD14, cKit+	(15)
Endothelial progenitor (EPC)	CD34+	(18)
Embryonic stem cells	CD31+/CD146+	(70, 71)
Induced pluripotent stem cells (iPSC)	CD31+/CD146+	(70, 72)
Umbilical cord blood (UCB)-derived myeloid progenitor cells	CD14+	(73)
Mesenchymal stem cells (MSC)	CD31−/CD34−/CD44+/CD45−/CD73+/CD90+/CD105+/CD140b+	(43, 45)

*A limited compilation of stem cells that are implicated in retinal diseases with specific emphasis on retinopathy where applicable*.

Recently, we and others have proposed the use of mesenchymal cells as an alternate approach to cell therapies for DR. Mesenchymal stem cells (MSC) that have been obtained from bone marrow, peripheral blood, cord blood as well as stem cells derived from other sources such as adipose tissue is expected to have an impact on cell therapy in eye diseases. For example, adult bone marrow MSC positive for CD90 have been shown to partially differentiate into photoreceptors *in vitro* and *in vivo* (32) and local, but not systemic, transplantation of MSC is neuroprotective in a rat glaucoma model (33). Recently, adipose stromal cells (ASC) transplanted into diseased corneas preserved transparency as well as differentiated into functional keratocytes suggesting that these cells could be used as a source for stromal regeneration and repopulation in diseased corneas (34). Differentiation potential of ASC into the neuroectodermal lineage, yielding cells with phenotypic characteristics of RPE cells also has been recently documented, which is expected to alleviate the non-exudative form of age-related macular degeneration (ARMD) characterized by a progressive decay of RPE cells at the posterior pole of the eye (35). In sodium iodate mediated acute retinal damage model, engineered MSC that overexpress neurotrophin-4 were found safe and very effective intravitreally suggesting engineered MSC may represent a useful strategy for treatment of different forms of retinopathies in the future (36).

## The Plasticity of Pericytes in Adipose Tissue

**Figure 1:**
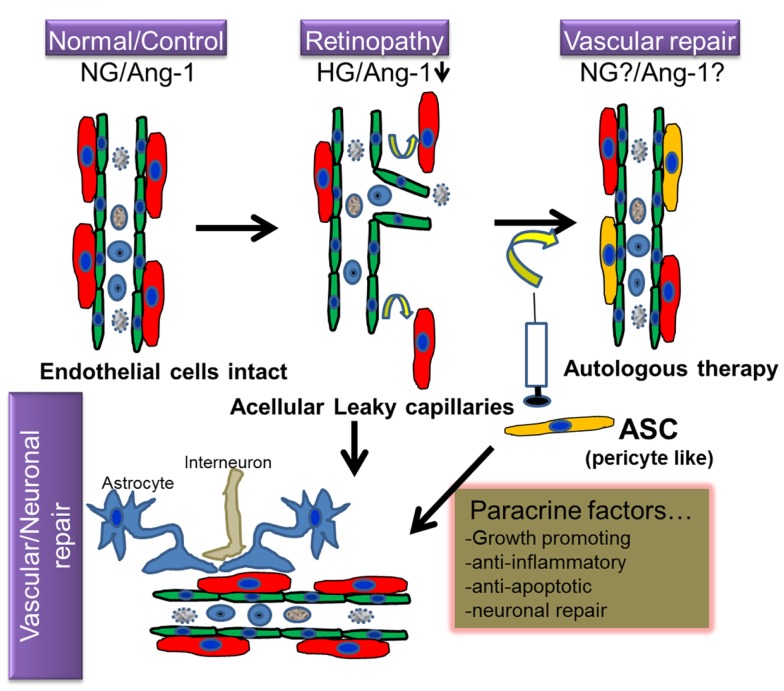
**Hypothetical model of regenerative cell therapy with adipose stromal cells (ASC) in retinopathy**. Under normoglycemic/non-pathological conditions (NG), intact retinal endothelium protected by pericytes is relatively impermeable; but becomes leaky under hyperglycemic stress (HG), in conjunction with downregulation of several proteins including Angiopoietin-1 (Ang-1), a characteristic feature observed in retinopathy. Because ASC and pericytes share phenotypic characteristics, it is hypothesized that cell therapy involving intravitreal injection of autologous ASC will ameliorate such loss of pericytes and consequent vascular permeability. Because ASC produce cytoprotective factors, it is anticipated that they will also promote vascular and neurodegeneration repair in retinopathy.

Adipose stromal cells are multipotential mesenchymal progenitor cells that are readily induced to undergo adipogenic differentiation, and have been recently demonstrated to have functional and phenotypic overlap with pericytes lining microvessels in adipose tissues (37, 38). Pericytes and ASC are both of mesenchymal origin and ASC can be differentiated into skeletal muscle cells, osteoblasts, chondrocytes, and adipocytes (39–42). Recent evidence suggests that human ASC and pericytes express identical surface markers including NG2, PDGFR α and β, and N-cadherin (37). Human adipose tissue sections revealed that ASC markers including both CD34 and CD140b were restricted to perivascular cells and formed robust functional vascular networks *in vivo* by cooperation of ASC with endothelial cells (37). The fact that ASC share functional properties as well as phenotypic markers with perivascular pericytes, makes these cells an attractive option to treat DR from the perspective of vascular stabilization and the restitution of pericyte dropout (Figure 1).

An elegant study by Yates group employed intravitreal injection of differentiated pericytes from human ASC via TGF-β1 treatment, could integrate into the retinal vasculature in two non-diabetic models, OIR and Akimba model on the abluminal locations around retinal capillaries, which is a defining characteristic of pericytes (43). Because the neovascular changes observed in the Akimba mouse are not due to long-term hyperglycemia, as in human DR (44), we developed a streptozotocin (STZ)-induced chronic hyperglycemia DR model and for the first time demonstrated that intravitreal injection of ASC in a chronic diabetes model pair with host vasculature in a perivascular location, possibly suggesting pericyte replacement (45). Interestingly, ASC requires a minimum of 3–6 weeks to pair with host vasculature to wrap around blood capillaries to stabilize vasculature *in vivo* as is also true with hind-limb ischemia models (46). Although more studies are warranted, the ability to provide such perivascular cells in the early stages of disease would represent a significant advancement in our understanding of the role of ASC cell therapy in DR.

Adipose stromal cells have a remarkable property to self-assemble into vascular structures in contact co-cultures with a number of endothelial cell types including retinal endothelial cells (45, 47). This property is not unique to ASC but also bone marrow MSC have been shown to form networks with HUVEC (48). It is quite interesting to note that MSC take up perivascular position while the endothelial cells form angiogenic tube like structures in this 2D coculture. These studies shed new light on the vasculogenic potential of ASC depends on interaction with endothelial cells involving contact and likely bi-directional interaction, resulting in modulated secretion of cytokines and matrix proteins that are essential for stabilized vessels. In support of this, recently ASC were shown to secrete Ang-1 in a time-dependent manner, especially when cultured in medium containing growth factors for vascular endothelial cells and promoted reendothelialization (49). Early data from our laboratory suggest that these vessel stabilizing properties of ASC are unaltered in hyperglycemic environment taking one step forward to suggest that ASC are pre-programed to sustain the hostile diabetic environment. More studies are warranted if ASC produced Ang-1 or other proteins play a vital role in the vascular stabilization and rescue from retinal injury.

## Mesenchymal Cells and Neuroprotection in DR

Apart from their role as perivascular cells, ASC are also known to produce a variety of angiogenic and anti-apoptotic factors (50), which in turn may promote dual beneficial effects addressing both capillary and neurodegeneration (Figure 1). We and others have shown that ASC act both in a paracrine manner as well as by direct physical interaction with endothelial cells to modulate angiogenesis (51), reduce skeletal muscle ischemia, and tissue loss (50), limit myocardial infarction (52), promote skin repair (53), and provide neuroprotective function against serum and potassium deprivation-induced cerebellar granule neuronal apoptosis (54, 55). These seminal studies have led to the concept that the adipose tissue may provide a novel autologous source of putative stem cells with significant potential for tissue repair and rescue from diabetic injury. The latter study establishes a mechanistic basis supporting the therapeutic application of ASC for neurological disorders, specifically through paracrine support provided by trophic factor secretion, which may be of paramount importance in regeneration of photoreceptors/astrocytes in retinal injury. To this end, intravenous injection of ASC in the STZ-induced DR rat model demonstrated an improvement in blood glucose levels as well as BRB integrity, with few donor cells differentiated into photoreceptor or astrocytes-like cells (56). Based on the fact that the diabetes itself is largely ameliorated with the intravenous injection of ASC via improved glucose tolerance, preserved beta cell mass, and increased beta cell proliferation in STZ-treated NOD-SCID mice (57), it is not clear if the improvement of BRB integrity is a direct result of ASC replacement of cells in the retina or due to the general decrease in blood glucose. Although the mechanisms are unclear, based on our studies performed with trophic factors in both STZ-induced DR and retinal ischemia–reperfusion (I/R) injury models, local intravitreal injection of trophic factors from ASC support the speculation that the paracrine trophic factors released by ASC play key roles by both stabilizing vasculature, and in protecting retinal cells from diabetic damage. In favor of this hypothesis, recently ASC have been shown to secrete physiologically relevant levels of several anti-apoptotic, anti-inflammatory, and chemotactic proteins (such as tumor necrosis factor-inducible gene 6, TSG6; stanniocalcin-1, STC-1; Rantes, CCL5; stem cell factor, SCF (37), tissue inhibitor of metalloproteinase1, TIMP-1 (57), which have been shown to mediate some of the beneficial effects of MSC (57–59). Future studies with detailed molecular approaches are needed to specifically identify the use of these specific proteins in DR.

## Clinical Trials in DR

At the time of this review, there are over 200 open clinical trials using MSC with diseases ranging from Crohn’s disease to cardiomyopathy to rheumatoid arthritis. There are only two clinical trials that directly address DR using bone marrow-derived MSC (NCT01518842) and bone marrow-derived CD34+ cells (a pilot clinical trial of the feasibility and safety of intravitreal autologous adult bone marrow stem cells in treating eyes with vision loss from retinopathy; NCT01736059). While MSC are not a preferred choice as of today, early 2012, FDA approved a first of its kind clinical trial (A phase I/II, open-label, multi-center, prospective study to determine the safety and tolerability of sub-retinal transplantation of human embryonic stem cell-derived retinal pigmented epithelial (MA09-hRPE) cells in patients with advanced dry AMD; NCT01344993) for the use of embryonic stem cells (ES) for the treatment of AMD, an age-related retinal disease with loss of central vision. It was reported that two patients who received stem cells showed improvement in their vision and it is encouraging that during the observation period of 4 months neither patient lost vision (60). However, ES cells are controversial and their use is tainted by ethical issues. Similarly, iPSC cells are being tested in Japan in a clinical study (61) and one can hope it will show enough efficacy to encourage formal clinical trials, however, as with ES, iPSC cells are also under scrutiny and the use of four genes to make a stem cell and their long-term efficacy is controversial. Obviously, safety and efficacy is one of the main key point for FDA approval, there are studies aiming at the very safety and efficacy of MSC in newly diagnosed Type 1 diabetic patients is ongoing, which will inform us on how these cells behave *in vivo* in long-term (Safety and efficacy of mesenchymal stem cells in newly diagnosed type 1 diabetic patients; NCT01322789). In particular, ASC are currently being investigated in clinical trials in several fields (chronic inflammation, ischemic diseases, etc.) and the emerging results of these trials will provide a great deal of data concerning the safety of ASC use (62). Unlike other stem cells such as iPSC, which needs extensive manipulation of cell armamentarium, ASC need few, if any, such manipulations making its use more advantageous over other cell types. Unfortunately, in countries like Mexico and Europe ASC are already being used in clinical treatment of DR, compromising the safety of patients as well as hindering our ability to perform rigorous clinical studies in US. We as a scientific community must perform rigorous scientific studies to ensure their safety without compromising the efficacy before offering clinical trials in the United States.

## Challenges and Future Directions of Cell Therapies in DR

There are multiple challenges for cell therapies with MSC in DR.

(1) Pericytes are mesenchymal in origin and express multiple cell surface markers (CD140b, CD146, NG2, SMA, Desmin) depending upon the tissue or the subpopulation derived from the tissues. This brings enormous heterogeneity in MSC, which may contribute to different outcomes in DR.
(2) The heterogeneity in MSC may mandate us to purify and enrich a subpopulation to define differentiation with specificity to pericyte. Emerging data from pericyte progenitors from MSC engraft preferentially at perivascular location (63, 64). Furthermore, precisely characterized pericytes are less likely to generate unwanted cell type making it an ideal candidate for FDA approval.
(3) Route of injection may be of concern. In line with other cell therapies approved for DR, we chose to do intravitreal injections of ASC as it benefits from direct delivery of these cells into the eye close to the damaged retinal vasculature. Furthermore, unlike for endothelial progenitor cells, pericytes from abluminal side may yield quick results. However, we would need to evaluate the relative merit of intravenous, intraorbital, or sub-retinal injection of MSC in future studies as these routes are routinely used in clinical ophthalmology.
(4) Well-characterized vasoregenerative cells that are ready for clinical application for human use is necessary. This may include a combination of MSC and CD34+ cells or other endothelial progenitor cells as one cell type alone may not help develop patented vessels. To this end, we have recently shown that ASC markedly enhanced retinal endothelial cell survival under hyperglycemic conditions; and in contact co-cultures, ASC formed robust vascular networks with retinal endothelial cells, much as with cord blood endothelial cells (45, 47). Emerging data indicate that direct cell–cell contact between MSC and endothelial cells may induce pericyte phenotype in MSC with marked expression of SMA as well as other pericyte markers such as CD146 (47, 65).
(5) One of the challenges for MSC transplantation therapy for DR is the secretion of angiogenic growth factors produced by ASC, such as VEGF and HGF (50), which may adversely affect retina for proliferative changes. Although MSC have been shown to produce paracrine trophic factors that may modulate between a pro-angiogenic to anti-angiogenic state depending upon the microenvironment (66–69), a careful regulation of ASC differentiation may be necessary.
(6) Another general challenge of stem cell-based treatments is the relative rudimentary characterization of derived cells. Most studies rely on a rather limited survey of extracellular markers rather than functional evaluation of the derived cells.

## Conclusion

Mesenchymal cells in particular ASC are preferred choices of cell therapy in ischemic and diabetic diseases. The ready availability of large numbers of autologous cells with the capabilities of facilitating repair of vascular and neurogenic tissues would be highly significant clinically for patients with retinal diseases such as AMD, DR, and retinitis pigmentosa. The resulting therapies would involve a minimally invasive procedure such as liposuction to harvest adipose tissue, followed by any processing required to obtain an appropriately defined and functionally consistent population of cells with the desired characteristics. Because of the extremely large numbers of cells available from such harvests, this processing might simply include isolation/separation steps in the absence of any need for expansion; or conversely might include culturing in the presence of either specific media, growth factors, or even genetic material that preferentially activate/suppress specific signaling events involved in competing differentiation pathways for the purpose of directing subsequent cellular behavior. Studies undertaken in rodent models will help determine if stem cells isolated from the adipose tissue can be used as vehicles for long-term, sustained delivery of therapeutic agents to eye tissues, or replace the diseased tissue with stem cells that can take their function. By evaluating this approach in the rodent model, we will be in a better position to determine whether such an approach should be tested in humans with a variety of retinal diseases.

## Conflict of Interest Statement

The Guest Associate Editor Ashay Dilip Bhatwadekar declares that, despite being affiliated to the same institution as author Gangaraju Rajashekhar, the review process was handled objectively and no conflict of interest exists. The author declares that the research was conducted in the absence of any commercial or financial relationships that could be construed as a potential conflict of interest.
